# The Effect of Light Therapy on Electroencephalographic Sleep in Sleep and Circadian Rhythm Disorders: A Scoping Review

**DOI:** 10.3390/clockssleep4030030

**Published:** 2022-08-09

**Authors:** Teha B. Pun, Craig L. Phillips, Nathaniel S. Marshall, Maria Comas, Camilla M. Hoyos, Angela L. D’Rozario, Delwyn J. Bartlett, Wendy Davis, Wenye Hu, Sharon L. Naismith, Sean Cain, Svetlana Postnova, Ron R. Grunstein, Christopher J. Gordon

**Affiliations:** 1 Faculty of Medicine and Health, The University of Sydney, Sydney, NSW 2050, Australia; 2 CIRUS, Centre for Sleep and Chronobiology, Woolcock Institute of Medical Research, Sydney, NSW 2037, Australia; 3 Faculty of Medicine, Health and Human Sciences, Macquarie University, Sydney, NSW 2109, Australia; 4 Healthy Brain Ageing Program, Brain and Mind Centre, The University of Sydney, Sydney, NSW 2050, Australia; 5 School of Psychology, Faculty of Science, The University of Sydney, Sydney, NSW 2050, Australia; 6 School of Architecture, Design and Planning, The University of Sydney, Sydney, NSW 2008, Australia; 7 School of Psychological Sciences and Turner Institute for Brain and Mental Health, Monash University, Melbourne, VIC 3800, Australia; 8 School of Physics, Faculty of Science, The University of Sydney, Sydney, NSW 2006, Australia; 9 Sleep and Severe Mental Illness Clinic, CPC-RPA Clinic, Royal Prince Alfred Hospital, Sydney, NSW 2050, Australia

**Keywords:** circadian rhythm disorder, electroencephalography, insomnia, light therapy, sleep disorder, quantitative EEG analysis

## Abstract

Light therapy is used to treat sleep and circadian rhythm disorders, yet there are limited studies on whether light therapy impacts electroencephalographic (EEG) activity during sleep. Therefore, we aimed to provide an overview of research studies that examined the effects of light therapy on sleep macro- and micro-architecture in populations with sleep and circadian rhythm disorders. We searched for randomized controlled trials that used light therapy and included EEG sleep measures using MEDLINE, PubMed, CINAHL, PsycINFO and Cochrane Central Register of Controlled Trials databases. Five articles met the inclusion criteria of patients with either insomnia or delayed sleep–wake phase disorder (DSWPD). These trials reported sleep macro-architecture outcomes using EEG or polysomnography. Three insomnia trials showed no effect of the timing or intensity of light therapy on total sleep time, wake after sleep onset, sleep efficiency and sleep stage duration compared to controls. Only one insomnia trial reported significantly higher sleep efficiency after evening light therapy (>4000 lx between 21:00–23:00 h) compared with afternoon light therapy (>4000 lx between 15:00–17:00 h). In the only DSWPD trial, six multiple sleep latency tests were conducted across the day (09:00 and 19:00 h) and bright light (2500 lx) significantly lengthened sleep latency in the morning (09:00 and 11:00 h) compared to control light (300 lx). None of the five trials reported any sleep micro-architecture measures. Overall, there was limited research about the effect of light therapy on EEG sleep measures, and studies were confined to patients with insomnia and DSWPD only. More research is needed to better understand whether lighting interventions in clinical populations affect sleep macro- and micro-architecture and objective sleep timing and quality.

## 1. Introduction

Light is the most potent *zeitgeber* for the entrainment of human circadian rhythms [1,2,3,4]. In the past, human circadian rhythms were clearly regulated by the periodic 24 h light/dark cycle of the sun [5,6], but the introduction of electric (artificial) light in modern industrialized societies over the last two centuries has significantly altered light exposure of the population [7,8]. Many people now receive lower levels of daytime bright light [9], due to longer periods of time being spent indoors, spending up to 90% of time per day under electric lights [10,11,12]. Additionally, people are also exposed to an increased amount of electric light at night [13]. Various characteristics of electric light sources (such as intensity, timing, duration and spectral composition) may disrupt circadian rhythms [14,15,16,17], with an individual’s master circadian oscillator in the brain, the suprachiasmatic nucleus (SCN), being particularly sensitive to short-wavelength light (peak around 480 nm) [15,18,19,20] and evening light exposure [21,22]. Whilst light therapies have been shown to have a small to medium effect on improving sleep and circadian rhythm disorders [23], the effect of electric light on brain neurophysiology assessed with electroencephalography (EEG) is still not fully understood.

Research has shown that increased exposure to light at night further compounds the adverse effects of inadequate daytime bright-light exposure [24,25,26,27]. In modern industrialized societies, the amount of electric light at night has been rising consistently at an average rate of 6 to 10% every year [13,28,29]. This potentially has a negative impact on circadian and sleep–wake physiology, as melatonin secretion and core body temperature rhythms are significantly altered with bright-light exposure at night [30,31,32], alongside increased sleep onset latency (SOL) [30,33,34] and decreased sleep quality [31,35]. Furthermore, the use of blue-enriched light-emitting electronic devices such as computers, tablets, televisions, mobile phones and video game consoles has risen significantly in the last two decades, with research showing that around 90–95% of individuals aged 13 to 64 years use an electronic device at least once per week prior to bedtime [36,37]. The use of electronic devices before bedtime is associated with delayed bedtime [37,38], longer SOL [39,40] and decreased total sleep time (TST) [39,41].

The potential for the intensity, spectral composition and timing of light exposure to alter sleep, both negatively and as a beneficial therapy, has prompted investigators to quantify its effects on objective sleep metrics. Intervention studies using light with various attributes have reported inconsistent effects on sleep in healthy individuals, night shift workers and patients with depression [42,43,44,45]. A systematic review found that light therapy was generally effective at reducing sleep problems, but the effect sizes were small to medium [23], and the analysis did not assess the effect on EEG-derived sleep measures. The majority of light therapy studies appear to have only examined the influence of the timing and composition of light on circadian variables or clinical symptoms and not on objective sleep. The accuracy of the subjective sleep assessment tools depends on an individual’s recall and perception of sleep [46]. Studies reported many discrepancies between subjective and objective sleep outcome measures [47,48]. Contrary to subjective sleep assessment tools, EEG directly quantifies brain activity, and the visual examination of EEG signals during sleep is commonly used to determine sleep stages [49,50,51] and diagnose sleep disorders [52]. EEG-derived sleep measures enable the detection of more fine-grain micro-architecture and sleep stage changes [53]. Objective sleep outcome measures can provide more insights into sleep quality and quantity that are not identified in subjective sleep outcome measures [48,54]. Furthermore, participants in light therapy studies might be aware of the intervention and control light conditions (intensity, timing, duration and spectral composition), which could have potentially impacted subjective sleep outcome measures. EEG-derived metrics are less likely to be affected by nocebo effects.

### 1.1. Rationale

To our knowledge, there are no existing systematic or scoping reviews examining the effect of light therapy on EEG-derived sleep in patients with sleep or circadian rhythm disorders. We used a scoping review, as we wanted to identify and map the existing research on light therapy and EEG-measured sleep.

### 1.2. Objective

The aim of this scoping review was to examine the effect of light therapy on sleep macro-architecture and micro-architecture (EEG spectral power density) in patients with sleep or circadian rhythm disorders in randomized controlled light intervention trials.

## 2. Methods

### 2.1. Protocol and Registration

We conducted this review using the methods of Arksey and O’Malley [55] and reported according to the Preferred Reporting Items for Systematic Reviews and Meta-Analyses extension for scoping reviews (PRISMA-ScR) [56]. A review protocol was not registered.

### 2.2. Eligibility Criteria

Studies with original data were included based on the following inclusion criteria, modelled on the PICOT format for an interventional question and specifying the study type (T).

1. Population: Participants with a sleep or circadian rhythm disorder.
2. Intervention: The intervention light therapy had to include either:
  - Intensity of light greater than or equal to control light condition;
  - Clock time (outside of regular light hours).

The study had to include any type of light therapy (daylight or electric) as a stand-alone treatment. If the study used light therapy as an adjunctive to other interventions such as sleep hygiene or caffeine, the non-light intervention component must have been used equally in the control and intervention groups.

3. Comparison: The control light condition had to include either:
  - Intensity of light less than or equal to intervention light condition;
  - Clock time (regular light hours).
4. Outcome: Sleep macro- or micro-architecture assessed with EEG or polysomnography recordings. Both nighttime and daytime sleep were included. Time in bed (TIB), TST, wake after sleep onset (WASO), sleep efficiency (SE), SOL and the duration of non-rapid eye movement (NREM) and rapid eye movement (REM) sleep were included as macro-architecture measures of sleep. Sleep micro-architecture was measured using EEG power spectral analysis, including any of the frequency bands (delta, theta, alpha, sigma and beta).
5. Study type: Laboratory or clinic-based studies where randomization had been used to assign participants to conditions (parallel trials) or the order in which they were exposed to conditions (cross-over trials).

### 2.3. Information Sources and Search

A search was carried out in five databases: MEDLINE, PubMed, Cumulative Index to Nursing and Allied Health Literature (CINAHL), Cochrane Central Register of Controlled Trials and PsycINFO. We searched for studies published from the inception date of the databases to May 2021. The following search terms were used to identify the relevant studies: (1) light therapy: “phototherapy” or “photo therapy” or “light exposure” or “light therapy” or “light treatment*” or “light intervention*” or “heliotherapy” or “bright light” or “blue light” or “white light” or “natural light” or “sunlight” or “polychromatic light” or “monochromatic light” or “artificial light” or “light”; (2) sleep and circadian rhythm disorders: “advanced sleep phase syndrome*” or “delayed sleep wake phase disorder*” or “delayed sleep phase syndrome*” or “circadian rhythm sleep disorder*” or “non 24 h sleep wake disorder*” or “shift work sleep disorder*” or “sleep wake cycle disorder*” or “shift work disorder*” or “insomnia*” or “early awakening” or “insomnia disorder*” or “nonorganic insomnia*” or “sleep initiation dysfunction*” or “transient insomnia*” or “jet lag syndrome” or “jet lag disorder*” or “jet lag” or “jetlag”; (3) sleep: “sleep*”. The search was restricted to articles published in the English language only. The search terms and strategies were adjusted depending on the database being used. The reference lists of the selected primary studies and past reviews were checked for any relevant papers that were not retrieved by our search strategy. The search syntax for each database is presented in Supplementary Material S1.

### 2.4. Selection of Sources of Evidence

All duplicates were removed from the initial article yield, and one author (TP) screened the titles and abstracts of all remaining articles against the inclusion/exclusion criteria. The remaining full-text articles were then screened, and excluded articles were assigned reasons for exclusion.

### 2.5. Data Charting Process and Data Items

Data extraction was performed by one author (TP) and recorded into a tabulated form. Relevant data included the study design, primary outcome, study location, sample size, participants (age, gender, diagnosis), characteristics of intervention and control lighting conditions, sleep EEG outcome measures and results. Extracted data were verified by an additional author (CP or CG). Any discrepancies in data extraction were discussed and resolved by consensus.

### 2.6. The Critical Appraisal of Individual Sources of Evidence

We did not conduct a critical appraisal of individual sources of evidence for this scoping review.

### 2.7. Synthesis of Results

Relevant information was recorded into the tabulated forms and was used to summarize and report the findings from different light therapy studies in patients with a sleep or circadian rhythm disorder. The standardized tabulated forms were useful for conducting a comparative analysis, identifying important themes from the data and synthesizing key elements.

## 3. Results

### 3.1. Selection of Sources of Evidence

**Figure 1:**
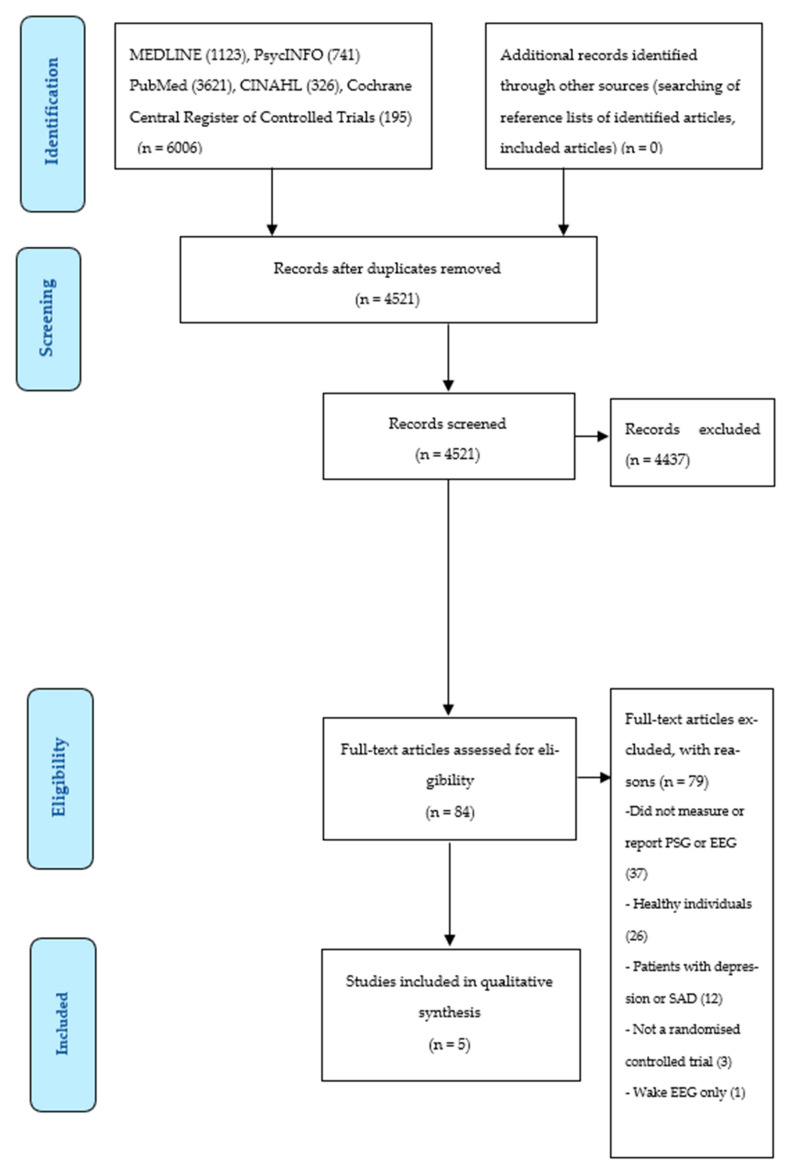
PRISMA flow diagram of search process and numerical outcomes. Abbreviations: EEG: electroencephalography, PSG: polysomnography, SAD: seasonal affective disorder.

The initial search yielded 6006 records from the databases: MEDLINE (n = 1123), PubMed (n = 3621), CINAHL (n = 326), PsycINFO (n = 741) and Cochrane Central Register of Controlled Trials (195). After duplicate records were removed, there were 4521 articles to review against the eligibility criteria. Following screening, 84 articles were subject to full-text screening. After reviewing the full-text articles, five studies met all eligibility criteria. Figure 1 shows the article selection flowchart.

### 3.2. Characteristics of Sources of Evidence

All five studies were conducted in the USA and published between 1990 and 2009 [57,58]. Four of the five studies were carried out using a randomized parallel-study design [58,59,60,61], with the remaining one being a randomized cross-over design [57]. The number of participants across all studies ranged from 7 to 102 [59,61]. Three studies enrolled older adults (mean age > 60 years) [58,59,60], and one study enrolled both young adults (age range = 20–40 years) and older adults (age range = 60–79 years) [61]. The age of participants was not reported in one study [57]. The duration of light administration for intervention and control groups varied from 45 min to 4 hours [58,61]. Light exposure sessions ranged from four days to three months. Most studies (four out of five) collected data during overnight sleep. Only one study carried out data collection during daytime multiple sleep latency tests [57]. The included studies incorporated two population groups: patients with insomnia and delayed sleep–wake phase disorder (DSWPD).

### 3.3. Results of Sources of Evidence

The characteristics of the included trials are outlined in Table 1. The effects of light therapy on EEG sleep measures are presented in Table 2.

### 3.4. Synthesis of Results

#### 3.4.1. Insomnia Studies

The relationship between the timing (afternoon versus evening) of bright-light exposure (>4000 lx) and sleep outcomes in older adults (≥60 years) with sleep maintenance insomnia was reported in two studies [59,60]. The evening light exposure group (21:00–23:00 h) had a significantly higher SE compared to the afternoon light exposure group (15:00–17:00 h) [60] (Table 2). In contrast, another study with an identical experimental protocol did not find any differences between the afternoon and evening light therapy exposure times for TIB, TST, WASO, SE, SOL and sleep stage duration [59]. The effect of light intensity (3000–4000 lx versus 1–65 lx) and timing of light exposure (early morning or daytime versus evening) in patients (>54 years) with insomnia and/or depression was reported in two studies [58,61], with no significant differences in sleep (TST, WASO, SE and sleep stage duration) reported.

#### 3.4.2. DWSPD Study

Only one study compared the effect of exposure to polychromatic light (2500 lx) for two hours in the early morning (06:00–09:00 h) combined with light restriction in the evening with exposure to polychromatic light (300 lx) for two hours in the early morning (06:00–09:00 h) on sleep latency in patients with DSWPD (Table 2) [57]. Six multiple sleep latency tests were performed during the day (09:00 and 19:00 h), and the study showed that administering polychromatic light (2500 lx) in the morning and restricting light in the evening significantly increased sleep latency in the morning (09:00 h and 11:00 h) [57].

#### 3.4.3. Sleep EEG Micro-Architecture

There were no studies that examined the impact of light therapy on sleep micro-architecture (power spectral analysis) in patients with a sleep or circadian rhythm disorder.

## 4. Discussion

This scoping review found only a small number of studies that examined the effect of light therapy on sleep macro-architecture in patients with a sleep or circadian rhythm disorder [57,58,59,60,61]. Studies were conducted in patients with insomnia [58,59,60,61] and DSWPD [57] only. No studies investigated the effect of light therapy on sleep micro-architecture using EEG power spectral analysis.

### 4.1. Summary of Evidence

#### 4.1.1. Patients with Insomnia

Our scoping review identified four light therapy studies in patients with insomnia. Three of those studies did not show any beneficial effects of light therapy on EEG sleep in patients with sleep maintenance or primary insomnia [58,59,61]. In contrast, only one study showed a positive effect of evening light therapy on SE in patients with sleep maintenance insomnia [60]. Lack et al. (1996) found that patients with sleep maintenance insomnia had significantly advanced circadian rhythms compared with healthy individuals [62]. Light therapy in the evening can delay the circadian rhythms of core body temperature and melatonin secretion [63,64]. Light therapy administered in the evening may re-establish a more normal phase relationship between circadian rhythms and sleep, resulting in higher SE in patients with sleep maintenance insomnia [60]. A possible explanation for inconsistent effects across studies could be variations in light therapy compositions (intensity, timing, duration and spectral composition), study settings (laboratory study versus field-based study), age-related structural changes in the visual and circadian systems, interindividual variations in light sensitivity, prior photic history and the endogenous period of the human circadian clock.

There are structural alterations in the visual and circadian systems associated with ageing [65,66,67]. Older adults may have higher ocular lens absorption [68], a smaller pupil size [69], lower lens transmittance [70] and a reduced number of circadian photoreceptors [65], resulting in reduced sensitivity to *zeitgebers*, particularly short-wavelength light [66,71]. The transmission of light from the eye to the SCN can be altered in older adults due to neurodegeneration of the SCN [67,72] and eye conditions such as glaucoma and macular degeneration [66]. These conditions may have impacted the outcomes due to the reduced photic input perceived by the SCN and/or the SCN being less responsive to light.

There is also a large interindividual variability in light sensitivity [22,73,74]. One recent study showed that individual variations in sensitivity to evening light for melatonin suppression are greater than 50 times [22]. Such a variation in light sensitivity plays a role in circadian and other physiological responses to light. However, none of the reviewed studies considered individual variations in sensitivity to light when designing light therapy for patients with insomnia. The sensitivity of the circadian system to light can also be influenced by prior photic history. For example, the administration of higher intensity of light during the day reduces melatonin suppression and circadian phase shift in response to light at night [75,76]. Conversely, exposure to dim light during the day increases melatonin suppression and circadian phase shifts induced by the evening or nighttime light exposure [77,78].

Interindividual differences in the endogenous period of the human circadian clock are due to variations of the different proteins (in terms of, e.g., levels, phosphorylation kinetics, degradation) that compose the molecular clock [79,80,81]. An individual with an intrinsic period longer than 24 h requires a daily phase advance in order to stay synchronized to the 24 h light/dark cycle. In contrast, an individual with an intrinsic period shorter than 24 h requires a daily phase delay. Individuals with shorter circadian periods tend to be more phase-delayed and less phase-advanced by the photic stimulus than individuals with longer circadian periods [82]. The efficacy of light therapy will also depend on the characteristics of each individual’s endogenous clock. However, none of the reviewed studies adjusted light therapy on an individual’s endogenous clock, which has likely contributed to the lack of noticeable effects of light therapy on electrophysiological sleep.

None of the reviewed studies considered spectral composition of light, which is critical in quantifying light acting on the circadian system via melanopsin-containing intrinsically photosensitive retinal ganglion cells [83,84]. Instead, they only reported photopic illuminance (lx), which quantifies the light affecting the visual system via rods and cones. The human circadian system is most sensitive to short-wavelength light (peaked around 480 nm), unlike the visual system with the peak sensitivity at 555 nm [20,83]. Thus, photopic illuminance (lx) does not allow the investigators to accurately compare the effect of lighting interventions with different spectral compositions in the human circadian system [83]. Future studies should measure and report melanopic illuminance (or melanopic irradiance) to allow comparison of the outcomes from different studies and replicating experimental designs [85].

A previous meta-analysis found a positive effect of light interventions on insomnia symptoms [23]. In contrast to our review, which only included randomized controlled studies with EEG-derived sleep outcomes, that meta-analysis included both randomized and non-randomized studies with subjective and objective sleep outcome measures (actigraphy or polysomnography) [23]. Further studies with larger sample sizes and robust study designs are required to determine the ideal intensity, duration, timing and spectral composition of light therapy for the treatment of insomnia.

#### 4.1.2. Patients with DSWPD

There was only one study that examined the effect of light therapy on DSWPD patients using daytime multiple sleep latency tests [57]. Bright light increased sleep latency by 4 to 5 min at 09:00 h and 11:00 h, in a within-arm analysis. However, this study did not report nocturnal sleep, had a small sample size (n = 15 for intervention light condition, n = 17 for control light condition) and was reported before clinical trial reporting guidelines and registrations were established. Typically, light therapy in DWSPD aims to phase shift the circadian clock. The administration of light therapy in the morning seems to be a reasonable and promising non-pharmacological intervention to advance the delayed sleep phase and improve sleep quality in patients with DSWPD. It should be noted that despite this limited empirical evidence, light therapy has been recognized as a treatment option for patients with DSWPD by the American Academy of Sleep Medicine [86]. Further interventional studies with robust study designs are required.

#### 4.1.3. Lack of Sleep EEG Micro-Architecture Outcomes

This scoping review could not find any research examining sleep EEG micro-architecture in sleep and circadian rhythm disorder patients. However, studies conducted in healthy individuals have reported that exposure prior to bedtime to blue monochromatic light (460 nm), blue-enriched polychromatic light (27.6 lx) or bright polychromatic light (2500 lx) compared with exposure to green monochromatic light (550 nm), and blue-depleted polychromatic light or dim light (6 lx) reduced delta activity (slow-wave activity) (0.75–4.5 Hz) during the first NREM sleep period [42,87,88] and increased delta activity in the third or fourth sleep cycle [42,87]. These findings suggest that low-intensity blue-enriched light prior to bedtime can negatively impact homeostatic sleep pressure by decreasing EEG spectral power during the early sleep period. However, other studies have not shown any effect of light therapy on the EEG power spectra once people without a sleep or circadian disorder fall asleep [40,89,90,91]. It is somewhat surprising that this has not been investigated to date in clinical populations, as the sleep EEG micro-architecture provides more insights into objective sleep quality that are not identified in macro-architectural sleep reports [92]. We recommend that studies should be conducted to determine the effects of light therapy on EEG spectral power density during sleep in patients with a sleep or circadian rhythm disorder.

### 4.2. Strengths and Limitations

The strengths of this scoping review are that this is the first review assessing the effect of light therapy on EEG-derived sleep measures in patients with sleep or circadian rhythm disorders and that it was carried out according to the PRISMA-ScR framework. This review found limited empirical evidence about the effect of light therapy on EEG sleep measures and highlighted the need for more research in this area. There are some limitations to this review. We limited our search to studies published in English. We also only examined sleep and circadian rhythm disorders, whereas there are studies examining the effect of light therapy on mental health outcomes [93,94,95], neurodegenerative diseases [96,97] and mild cognitive impairment [98]. However, these were not focuses of this review.

## 5. Conclusions

Overall, the effect of light therapy on EEG sleep measures in patients with a sleep or circadian rhythm disorder is still an understudied area of research. We could not draw firm conclusions on the effects of light therapy on sleep macro-architecture in patients with insomnia due to a lack of consistent findings across studies. There were no studies that examined the effect of light therapy on sleep micro-architecture (power spectral analysis) in patients with a sleep or circadian rhythm disorder. Well-designed and adequately powered studies are required to determine an effective mixture of intensity, spectral composition, duration and timing of light therapy for sleep macro and micro-architecture measures in different clinical populations. Such studies will provide more consistent evidence on which to base effective light treatments.

## Figures and Tables

**Table 1:** Characteristics of randomized studies comparing the effect of light therapy on macro-architecture of sleep EEG.

Patients with Delayed Sleep Phase Syndrome and Insomnia								
Authors, Date, Country	Design	Primary Outcome	Sample Size Sex	Age (Mean ± SD, Range)	Population	Intervention Light	Control Light	Sleep Macro-Architecture Variable Reported
Rosenthal et al. (1990), USA	Cross-over RCT Duration: 2 weeks	MSLT, core body temperature	I: 15, C: 17, sex not specified	NR	Delayed sleep phase syndrome	Illuminance: 2500 lx Clock time: 06:00–09:00 h for 2 h CCT: NR Wavelength: NR	Illuminance: 300 lx Clock time: 06:00–09:00 h for 2 h CCT: NR Wavelength: NR	SOL
Murphy and Campbell (1996), USA	Parallel pseudo-RCT Duration: Twice per week for 3 months	Core body temperature and performance tasks	8 F, 8 M (13 completers)	73.1 y, (60–82 y)	Insomnia	Illuminance: >4000 lx Clock time: 21:00–23:00 CCT: NR Wavelength: NR	Illuminance: >4000 lx Clock time: 15:00–17:00 CCT: NR Wavelength: NR	SE (%) -Averaged 2 nights
Suhner et al. (2002), USA	Parallel RCT Duration: Twice per week for 3 months	Sleep EEG and core body temperature	7 F, 8 M (I: 9 completers, C: 5 completers)	71.5 y, (63–84 y)	Insomnia	Illuminance: >4000 lx Clock time: 21:00–23:00 CCT: NR Wavelength: NR	Illuminance: >4000 lx Clock time: 15:00–17:00 CCT: NR Wavelength: NR	TIB (min) TST (min) WASO (min) SE (%)SOL (min) S1 (% TST) S2 (% TST) S3 (% TST) S4 (% TST) REM (% TST) -Averaged 2 nights
Youngstedt et al. (2005), USA	Parallel RCT Duration: 4 days for each condition	Mood, sleep EEG and melatonin	49 F, 23 M (older adults) 15 F, 15 M (young adults)	(60–79 y) (Older adults) (20–40 y) (Young adults)	Insomnia and/or depression Healthy	Illuminance: 3000 lx Clock time: Intervention 1: 1–3 h after awakening and 2 h before bedtime Intervention 2: 6–10 h after awakening CCT: NR Wavelength: NR	Illuminance: 1 lx Clock time: 6–10 h after awakening CCT: NR Wavelength: NR	TST ^†^ WASO ^†^ SE ^†^ -Averaged 4 nights
Friedman et al. (2009), USA	Parallel RCT, single-blinded Duration: 12 weeks	Sleep EEG and melatonin	36 F, 25 M (49 completers)	63.6 ± 7.1 y, (54–78 y)	Insomnia	Illuminance: ~4000 lx Clock time: Intervention 1: 15 min after awakening for 45 min Intervention 2: 1 h before bedtime for 45 min CCT: NRWavelength: NR	Illuminance: ~65 lx Clock time: Control 1: 15 min after awakening for 45 min Control 2: 1 h before bedtime for 45 min CCT: NR Wavelength: NR	TIB (min) TST (min) WASO (min) SE (%) S1 (%) S2 (%) S3 (%) S4 (%) REM (%) -Averaged 2 nights

*Abbreviations:* C: control; CCT: correlated color temperature; EEG: electroencephalography; F: females; I: intervention; M: males; MSLT: multiple sleep latency test; NR: not reported; RCT: randomized controlled trial; REM: rapid eye movement sleep stage; S1: stage 1 sleep according to the criteria of Rechtschaffen and Kales (1968); S2: stage 2 sleep according to the criteria of Rechtschaffen and Kales (1968); S3: stage 3 sleep according to the criteria of Rechtschaffen and Kales (1968); S4: stage 4 sleep according to the criteria of Rechtschaffen and Kales (1968); SD: standard deviation; SE: sleep efficiency; SOL: sleep onset latency; TIB: time in bed; TST: total sleep time; WASO: wake after sleep onset; y: year. *Key:* ^†^: outcomes were not numerically quantified

**Table 2:** Differences in macro-architecture of sleep EEG measures in light therapy conditions compared to control light conditions.

Patients with Delayed Sleep Phase Syndrome and Insomnia							
Author/s, Date	*Macro-Architecture Measures*						
	TIB	TST	WASO	SE	SOL	NREM	REM
Rosenthal et al. (1990) o					↑		
Murphy and Campbell (1996) •				↑			
Suhner et al. (2002) •	ns	ns	ns	ns	ns		ns
Youngstedt et al. (2005) •		ns	ns	ns			
Friedman et al. (2009) •	ns	ns	ns	ns			ns

*Abbreviations:* NREM: non-rapid eye movement sleep stage; REM: rapid eye movement sleep stage; SE: sleep efficiency; SOL: sleep onset latency; TIB: time in bed; TST: total sleep time; WASO: wake after sleep onset. Key: upwards arrow denotes a statistically significant increase in sleep EEG measure in intervention light conditions compared with control light conditions; downwards arrow denotes a statistically significant decrease in sleep EEG measure in intervention light conditions compared with control light conditions; ns: no significant difference between intervention and control light conditions; **•**: nighttime sleep EEG measure; o: daytime sleep EEG measure.

## Data Availability

Not applicable.

## Supplementary Materials

The following are available online at https://www.mdpi.com/article/10.3390/clockssleep4030030/s1, S1: Search strategy.

## Abbreviations

CINAHL	Cumulative Index to Nursing and Allied Health Literature
DSWPD	Delayed sleep–wake phase disorder
EEG	Electroencephalography
NREM	Non-rapid eye movement
PRISMA-ScR	Preferred Reporting Items for Systematic Reviews and Meta-Analyses extension for scoping reviews
REM	Rapid eye movement
SCN	Suprachiasmatic nucleus
SE	Sleep efficiency
SOL	Sleep onset latency
TIB	Time in bed
TST	Total sleep time
WASO	Wake after sleep onset
