# Supplementary material for: The Effect of Light Therapy on Electroencephalographic Sleep in Sleep and Circadian Rhythm Disorders: A Scoping Review
Source: Clocks Sleep. 2022 Aug 9;4(3):358–73. doi: 10.3390/clockssleep4030030 (PMC9397048; doi:10.3390/clockssleep4030030)
Supplement: Supplementary file 1 [file clockssleep-04-00030-s001.zip › clockssleep-1710889-supplementary.pdf]

Supplementary Materials S1

Search Strategy

Databases search alphabetically
CINAHL (Cumulative Index to Nursing and Allied Health Literature)
Cochrane Central Register of Controlled Trials
MEDLINE
PsycINFO
PubMed

Keywords and controlled vocabulary
Both keywords and controlled vocabulary were used as appropriate to conduct the literature searches. The search strategies for all databases are shown below.

<u>CINAHL</u>

S1. (MH "Phototherapy+")
S2. Phototherapy
S3. "photo therapy"
S4. "light therapy"
S5. "light exposure"
S6. "light treatment*"
S7. "light intervention*"
S8. Heliotherapy
S9. "bright light"
S10. "blue light"
S11. "white light"
S12. "natural light"
S13. (MH "Sunlight+")
S14. Sunlight
S15. "polychromatic light"
S16. "monochromatic light"
S17. "artificial light"
S18. "light"
S19. S1 OR S2 OR S3 OR S4 OR S5 OR S6 OR S7 OR S8 OR S9 OR S10 OR S11 OR S12 OR S13 OR S14 OR S15 OR S16 OR S17 OR S18
S20. (MH "Sleep Disorders, Circadian Rhythm+")
S21. "advanced sleep phase syndrome*"
S22. "delayed sleep wake phase disorder*"
S23. "delayed sleep phase syndrome*"
S24. "circadian rhythm sleep disorder*"
S25. "non 24 h sleep wake disorder*"
S26. "shift work sleep disorder*"
S27. "sleep wake cycle disorder*"
S28. "shift work disorder*"
S29. "sleep-wake schedule disorder*"
S30. (MH "Insomnia")
S31. "early awakening"
S32. "insomnia disorder*"
S33. "nonorganic insomnia*"
S34. "sleep initiation dysfunction*"
S35. "transient insomnia*"
S36. (MH "Jet Lag Syndrome")
S37. "jet lag disorder*"

S38. “jet lag*”
S39. “jetlag*”
S40. S21 OR S22 OR S23 OR S24 OR S25 OR S26 OR S27 OR S28 OR S29 OR S30 OR S31 OR S32 OR S33 OR S34 OR S35 OR S36 OR S37 OR S38 OR S39
S41. (MH “Sleep”)
S42. “sleep*”
S43. S41 OR S42
S44. S19 AND S40 AND S43
S45. S43, Limiters-English Language

<u>Cochrane Central Register of Controlled Trials</u>

1. exp phototherapy/
2. phototherapy.mp.
3. photo therapy.mp.
4. light therapy.mp.
5. light exposure.mp.
6. light treatment*.mp.
7. light intervention*.mp.
8. heliotherapy.mp.
9. bright light.mp.
10. blue light.mp.
11. white light.mp.
12. natural light.mp.
13. sunlight/
14. sunlight.mp.
15. polychromatic light.mp.
16. monochromatic light.mp.
17. artificial light.mp.
18. light.mp
19. 1 or 2 or 3 or 4 or 5 or 6 or 7 or 8 or 9 or 10 or 11 or 12 or 13 or 14 or 15 or 16 or 17 or 18
20. Sleep Disorders, Circadian Rhythm/
21. advanced sleep phase syndrome*.ti,ab.
22. delayed sleep wake phase disorder*.ti,ab.
23. delayed sleep-wake phase disorder*.ti,ab.
24. delayed sleep phase syndrome*.ti,ab.
25. non 24 h sleep wake disorder*.ti,ab.
26. shift work sleep disorder*.ti,ab.
27. shift work disorder*.ti,ab.
28. sleep wake cycle disorder*.ti,ab.
29. sleep-wake schedule disorder*.ti,ab.
30. “Sleep Initiation and Maintenance Disorders”/
31. insomnia*.ti,ab.
32. early awakening.ti,ab.
33. nonorganic insomnia*.ti,ab.
34. sleep initiation dysfunction*.ti,ab.
35. transient insomnia*.ti,ab.
36. Jet Lag Syndrome/
37. jet lag disorder*.ti,ab.
38. jet lag*.ti,ab.
39. jetlag*.ti,ab.
40. 20 or 21 or 22 or 23 or 24 or 25 or 26 or 27 or 28 or 29 or 31 or 32 or 33 or 34 or 35 or 36 or 37 or 38 or 39

41. Sleep/
42. sleep*.ti,ab.
43. 41 or 42
44. 19 and 40 and 43
45. Limit 40 to English language

MEDLINE

1. exp phototherapy/
2. phototherapy.mp.
3. photo therapy.mp.
4. light therapy.mp.
5. light exposure.mp.
6. light treatment*.mp.
7. light intervention*.mp.
8. heliotherapy.mp.
9. bright light.mp.
10. blue light.mp.
11. white light.mp.
12. natural light.mp.
13. sunlight/
14. sunlight.mp.
15. polychromatic light.mp.
16. monochromatic light.mp.
17. artificial light.mp.
18. light.mp
19. 1 or 2 or 3 or 4 or 5 or 6 or 7 or 8 or 9 or 10 or 11 or 12 or 13 or 14 or 15 or 16 or 17 or 18
20. Sleep Disorders, Circadian Rhythm/
21. advanced sleep phase syndrome*.ti,ab.
22. delayed sleep wake phase disorder*.ti,ab.
23. delayed sleep-wake phase disorder*.ti,ab.
24. delayed sleep phase syndrome*.ti,ab.
25. non 24 h sleep wake disorder*.ti,ab.
26. shift work sleep disorder*.ti,ab.
27. shift work disorder*.ti,ab.
28. sleep wake cycle disorder*.ti,ab.
29. sleep-wake schedule disorder*.ti,ab.
30. "Sleep Initiation and Maintenance Disorders"/
31. insomnia*.ti,ab.
32. early awakening.ti,ab.
33. nonorganic insomnia*.ti,ab.
34. sleep initiation dysfunction*.ti,ab.
35. transient insomnia*.ti,ab.
36. Jet Lag Syndrome/
37. jet lag disorder*.ti,ab.
38. jet lag*.ti,ab.
39. jetlag*.ti,ab.
40. 20 or 21 or 22 or 23 or 24 or 25 or 26 or 27 or 28 or 29 or 31 or 32 or 33 or 34 or 35 or 36 or 37 or 38 or 39
    Sleep/
41. sleep*.ti,ab.
42. 41 or 42
43. 19 and 40 and 43
44. Limit 40 to English language

PsycINFO

1. exp phototherapy/
2. phototherapy.mp.
3. photo therapy.mp.
4. light therapy.mp.
5. light exposure.mp.
6. light treatment*.mp.
7. light intervention*.mp.
8. heliotherapy.mp.
9. bright light.mp.
10. blue light.mp.
11. white light.mp.
12. natural light.mp.
13. sunlight/
14. sunlight.mp.
15. polychromatic light.mp.
16. monochromatic light.mp.
17. artificial light.mp.
18. light.mp
19. 1 or 2 or 3 or 4 or 5 or 6 or 7 or 8 or 9 or 10 or 11 or 12 or 13 or 14 or 15 or 16 or 17 or 18
20. Sleep Disorders, Circadian Rhythm/
21. advanced sleep phase syndrome*.ti,ab.
22. delayed sleep wake phase disorder*.ti,ab.
23. delayed sleep-wake phase disorder*.ti,ab.
24. delayed sleep phase syndrome*.ti,ab.
25. non 24 h sleep wake disorder*.ti,ab.
26. shift work sleep disorder*.ti,ab.
27. shift work disorder*.ti,ab.
28. sleep wake cycle disorder*.ti,ab.
29. sleep-wake schedule disorder*.ti,ab.
30. “Sleep Initiation and Maintenance Disorders”/
31. insomnia*.ti,ab.
32. early awakening.ti,ab.
33. nonorganic insomnia*.ti,ab.
34. sleep initiation dysfunction*.ti,ab.
35. transient insomnia*.ti,ab.
36. Jet Lag Syndrome/
37. jet lag disorder*.ti,ab.
38. jet lag*.ti,ab.
39. jetlag*.ti,ab.
40. 20 or 21 or 22 or 23 or 24 or 25 or 26 or 27 or 28 or 29 or 31 or 32 or 33 or 34 or 35 or 36 or 37 or 38 or 39
41. Sleep/
42. sleep*.ti,ab.
43. 41 or 42
44. 19 and 40 and 43
45. Limit 40 to English language

PubMed

1. ((((((((((((((((((phototherapy) OR (Phototherapy[MeSH Terms])) OR (“photo therapy”)) OR (“light therapy”)) OR (“light exposure”)) OR (“light treatment”)) OR (“light intervention”)) OR (heliotherapy)) OR (“bright light”)) OR (“blue light”)) OR (“white light”)) OR (“natural light”)) OR (sunlight)) OR (sunlight[MeSH Terms])) OR (“polychromatic light”)) OR (“monochromatic light”)) OR (“artificial light”)) OR (light)) AND (((((((((((((sleep disorder[MeSH Terms]) OR (circadian rhythm disorder[MeSH Terms])) OR (advanced sleep phase syndrome)) OR (delayed sleep wake phase disorder)) OR (circadian rhythm sleep disorder)) OR (delayed sleep phase syndrome)) OR (non 24 h sleep wake disorder)) OR (shift work sleep disorder)) OR (shift work disorder)) OR (sleep wake cycle disorder)) OR (sleep-wake schedule disorder)) OR (((((((insomnia[MeSH Terms]) OR (insomnia)) OR (early awakening)) OR (insomnia disorder)) OR (nonorganic insomnia)) OR (sleep initiation dysfunction)) OR (transient insomnia))) OR (((Jet Lag Syndrome[MeSH Terms]) OR (jet lag disorder)) OR (jetlag disorder)))) AND ((sleep[MeSH Terms]) OR (sleep)) Filters: English.
